# Functional and structural analysis of catabolite control protein C that responds to citrate

**DOI:** 10.1038/s41598-021-99552-x

**Published:** 2021-10-13

**Authors:** Wei Liu, Jinli Chen, Liming Jin, Zi-Yong Liu, Ming Lu, Ge Jiang, Qing Yang, Chunshan Quan, Ki Hyun Nam, Yongbin Xu

**Affiliations:** 1 Department of Bioengineering, College of Life Science, Dalian Minzu University, Dalian, 116600 Liaoning China; 2 Key Laboratory of Biotechnology and Bioresources Utilization of Ministry of Education, College of Life Science, Dalian Minzu University, Dalian, China; 3 School of Life Science and Biotechnology, Dalian University of Technology, No. 2 Linggong Road, Dalian, 116024 Liaoning China; 4 Shandong Provincial Key Laboratory of Energy Genetics, Key Laboratory of Biofuel, Qingdao Institute of Bioenergy and Bioprocess Technology, Chinese Academy of Sciences, Qingdao, 266101 Shandong China; 5 School of Life Science and Biotechnology, Dalian University, Dalian, 116622 Liaoning China; 6 Department of Life Science, Pohang University of Science and Technology, Pohang, 35398 Republic of Korea

**Keywords:** X-ray crystallography, Molecular biology

## Abstract

Catabolite control protein C (CcpC) belongs to the LysR-type transcriptional regulator (LTTR) family, which regulates the transcription of genes encoding the tricarboxylic acid branch enzymes of the TCA cycle by responding to a pathway-specific metabolite, citrate. The biological function of CcpC has been characterized several times, but the structural basis for the molecular function of CcpC remains elusive. Here, we report the characterization of a full-length CcpC from *Bacillus amyloliquefaciens* (BaCcpC-FL) and a crystal structure of the C-terminal inducer-binding domain (IBD) complexed with citrate. BaCcpC required both dyad symmetric regions I and II to recognize the *citB* promoter, and the presence of citrate reduced *citB* promoter binding. The crystal structure of CcpC-IBD shows two subdomains, IBD-I and IBD-II, and a citrate molecule buried between them. Ile100, two arginines (Arg147 and Arg260), and three serines (Ser129, Ser189, and Ser191) exhibit strong hydrogen-bond interactions with citrate molecules. A structural comparison of BaCcpC-IBD with its homologues showed that they share the same tail-to-tail dimer alignment, but the dimeric interface and the rotation between these molecules exhibit significant differences. Taken together, our results provide a framework for understanding the mechanism underlying the functional divergence of the CcpC protein.

## Introduction

The tricarboxylic acid (TCA) cycle, also known as the Krebs cycle or the citric acid cycle (CAC), is a central metabolic pathway in the cell^1^. The TCA cycle provides organisms with reducing potential, energy, and three of the 13 biosynthetic intermediates^2^. In *Bacillus*, TCA activity is controlled by several important regulatory proteins, including global regulators catabolite control protein A (CcpA) and CodY and the specific regulator catabolite control protein C (CcpC), which are coordinated by fructose-1,6-bisphosphate (FBP) and glucose-6-phosphate, guanosine-5′-triphosphate (GTP) and branched-chain amino acids (BCAAs), and citrate, respectively^3^. CcpA and CodY are metabolite-responsive global regulators of carbon metabolism pathways^4^. These global regulators coordinate the expression of numerous metabolic, biosynthetic and virulence genes that respond to three metabolites^5^. CcpA is a member of the LacI/GalR family of transcriptional repressors, which exert both direct (through citrate synthase, *citZ,* and *ccpC*) and indirect effects on TCA branch enzyme expression^6^. CodY is also a repressor of the *citB* gene belonging to a unique family of regulators in *B. subtilis* and other homologues of gram-positive bacteria^7^. Recent studies have shown that CcpC and CcpE exclusively regulate the TCA branch enzymes of the TCA cycle (*citB*, aconitase; *citC*, isocitrate dehydrogenase; and *citZ,* citrate synthase) by responding to a pathway-specific metabolite for both *Bacillus subtilis* and *Staphylococcus aureus*, respectively^8,9^.

CcpC widely exists in prokaryotes and is classified as belonging to the LysR-type transcriptional regulator (LTTR) family^10^. Typical LTTR family proteins comprise approximately 330 amino acids that form structures highly similar to those of N-terminal DNA-binding domains (DBDs), which are directly involved in DNA interactions, and poorly conserved C-terminal inducer-binding domains (IBDs) and are known to adopt different oligomeric states^10^. The DBD is highly conserved and directly involved in DNA interactions, similar to the helix-loop-helix, zinc finger, and β–sheet-anti-parallel domains^11–13^. IBD serves as a binding site of inducer and thus plays an important role in transcriptional activation by binding to inducers^14^.

To reveal the molecular mechanism of LTTRs, the structure of LTTRs has long been studied. The crystal structure of the DBD in complex with recognition binding site (RBS) has been determined in BenM, which revealed that 25-bp DBD of BenM interacting with *benA*-RBS^15^. While the crystal structure of BenM-DBD complexed RBS showed the details of the interaction, the mechanism responsible for the selection of a specific sequence remains elusive^15^. The RBS of BenM and CbnR have high similarity^16^. The crystal structure of the DBD of CbnR complexed with RBS reveals the detailed mechanism of the specific interaction between CbnR and its promoter DNA and that Thr33 leads to selective interactions with DNA^16^. Moreover, structural information of the IBD of LTTRs obtained is equally necessary. The first crystal structure of LTTRs is IBD of CysB, and the IBD induces conformational changes, resulting in structural changes for transcriptional activation^17^. Therefore, the mechanism of the conformational change plays an important role in the study of LTTRs^17^. The conformational change of IBD upon inducer binding has been structurally analysed in AphB, BenM, CcpE, and OxyR^18–21^. While several models of transcriptional activation of LTTRs with significant conformational changes have been proposed, all structural changes seem to be caused by the IBD upon inducer binding^18,22,23^. The first full-length crystal structure of CbnR, which exists in the crystal structure as a tetramer, was determined^24^. Four DBDs are located at the bottom of the tetramer of CbnR and arranged in a V-shape, which provides significant insight into DNA bending^24^. Small angle X-ray scattering (SAXS) studies of DntR have provided clear evidence of the structural changes that the LTTR tetramer undergoes upon activation via an inducer, and have supported the ‘sliding dimer’ hypothesis concerning LTTR transcription activation mechanisms^22^. The crystal structure of the full-length CbnR complex with its promoter DNA showed the mechanism of the quaternary structural change caused by inducer binding^14^. These changes are likely to be necessary for recruiting RNA polymerase to the promoter site to initiate transcription^25^.

In *B. subtilis*, CcpC binds to two sites within the *citB* promoter region, a dyad symmetry element centred at position −66 that induces of the transcriptional start site and a half-dyad element located at position −27 to −33^26^. Two CcpC dimers interact with these sites for repression, resulting in bending of the DNA, and then blocking the access of RNA polymerase to the promoter, which results in repression of *citB* expression. As with other LTTR proteins, CcpC interacts with the inducer to relieve this repression; that is, citrate induces the expression of *citB*^26^. The coinducer citrate is important for the function of CcpC and appears to function as a key catabolite for coordinating the *B. subtilis* metabolic state by binding to and activating CcpC^9^. Previous studies have indicated citrate-induced derepression in the *citB* gene of *L. monocytogenes*. *L. monocytogenes* CcpC represses the transcription of *citB* and *citZ*^27^. The binding site of the *citB* regulatory region in *L. monocytogenes* CcpC is almost consistent that in *B. subtilis* CcpC, with a dyad symmetry element centred at positions −68 to −28^27^. In addition, binding to the full dyad is maintained, however, binding to the half-dyad is reduced in the presence of citrate^27^. Thus, the CcpC complex with citrate is a signal that morphs CcpC into a conformation that is competent for binding DNA and inducing gene transcription. Several biological functional properties of CcpC are well characterized; however, the structure-based molecular function has been elusive^9^.

In this study, we characterized the full-length *Bacillus amyloliquefaciens* CcpC and determined the crystal structure of the C-terminal IBD of *B. amyloliquefaciens* CcpC (BaCcpC-IBD) at 2.3 Å resolution. The *citB* binding properties and the oligomeric state of BaCcpC were analysed. The crystal structure of BaCcpC-IBD was compared with structures of the LTTR family members. Taken together, our findings provide insight into the citrate-responsive mechanism of CcpC.

## Results and discussion

### Biochemical study of BaCcpC

**Figure 1:**
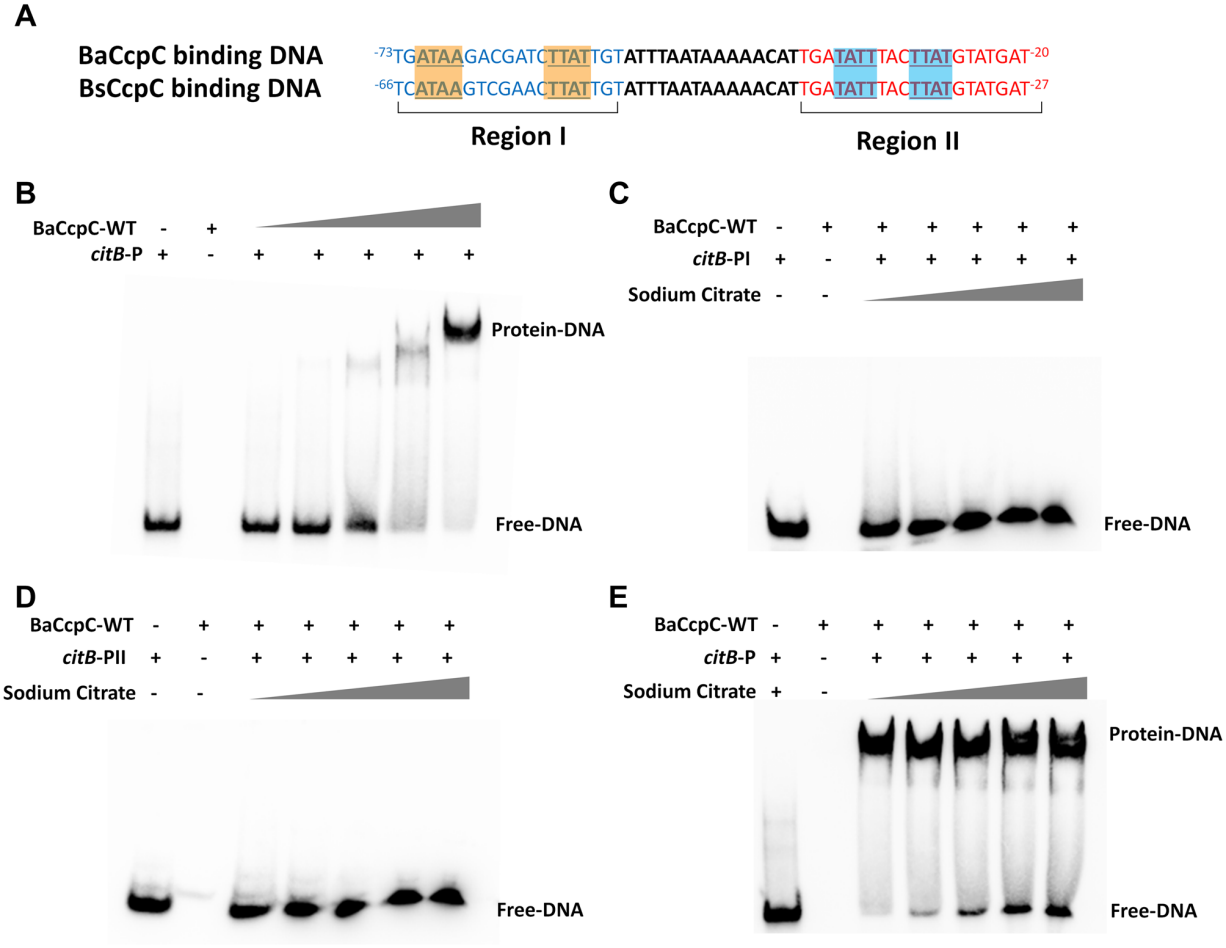
*citB* promoter-binding properties of BaCcpC. (**A**) Comparison of the *citB* promoter regions from *B. amyloliquefaciens* (upper) and *B. subtilis* (bottom). DNA-binding boxes are indicated in bold and underlined. EMSA experiment of BaCcpC with (**B**) the *citB* promoter, (**C**) region I in the *citB* promoter region, (**D**) region II in the *citB* promoter region, and (**E**) the *citB* promoter with various concentrations of sodium citrate.

In *B. subtilis*, CcpC (BsCcpC) negatively regulates *citB* gene expression, which is responsible for the interconversion of citrate and isocitrate^9^. The BsCcpC binding region forms two dyad symmetry elements centred at positions −66 and −27 (Fig. 1A)^9^. This BsCcpC bind to the DNA-binding boxes “ATAA”, “TTAT”, and “TATT” in the *citB* promoter region^9^. In the *B. amyloliquefaciens* genome, the potential promoter region of *citB* (named *citB*-P) was found from position −73 to −20, and it shows high similarity with the same DNA-binding boxes “ATAA”, “TTAT”, and “TATT” (Fig. 1A). The DNA sequence of the BsCcpC binding box was identical to that of the BaCcpC binding box in *citB* promoter region I (named *citB*-PI, −73 to −54), but the nonbinding sequences did not match, whereas the DNA sequences of *citB* promoter region II (named *citB*-PII, −40 to −20) matched a consensus sequence (Fig. 1A). Moreover, the spacer sequence between *citB* protomer regions I and II was identical to those of *B. subtilis* and *B. amyloliquefaciens*, and the same finding was obtained for the DNA length*.*

To verify whether CcpC regulates the predicted *citB* promoter region in *B. amyloliquefaciens*, we performed an electrophoretic mobility shift assay (EMSA) using a 56 bp *citB* promoter DNA fragment (from nucleotides −73 to −20 relative to the start codon of *citB*) as a probe (Fig. 1A). A complete shift of the free probe was observed as the concentration of BaCcpC increased (Fig. 1B). This result indicates that BaCcpC has a high binding affinity for *citB* promoter DNA of *B. amyloliquefaciens*. Next, the binding of BaCcpC to each of the *citB* promoter regions (I and II) for each CcpC was assessed. BaCcpC did not bind to either promoter region (Fig. 1C, D). These results indicate that both dyad symmetry promoter regions are required for BaCcpC to bind to the promoter of *citB*. To determine whether citrate affects the binding of CcpC to the *citB* promoter, an EMSA of CcpC for *citB* was performed with citrate. The results showed that the presence of citrate slightly suppressed the binding of CcpC to *citB*-P (Fig. 1E).

### Oligomerization of BaCcpC

**Figure 2:**
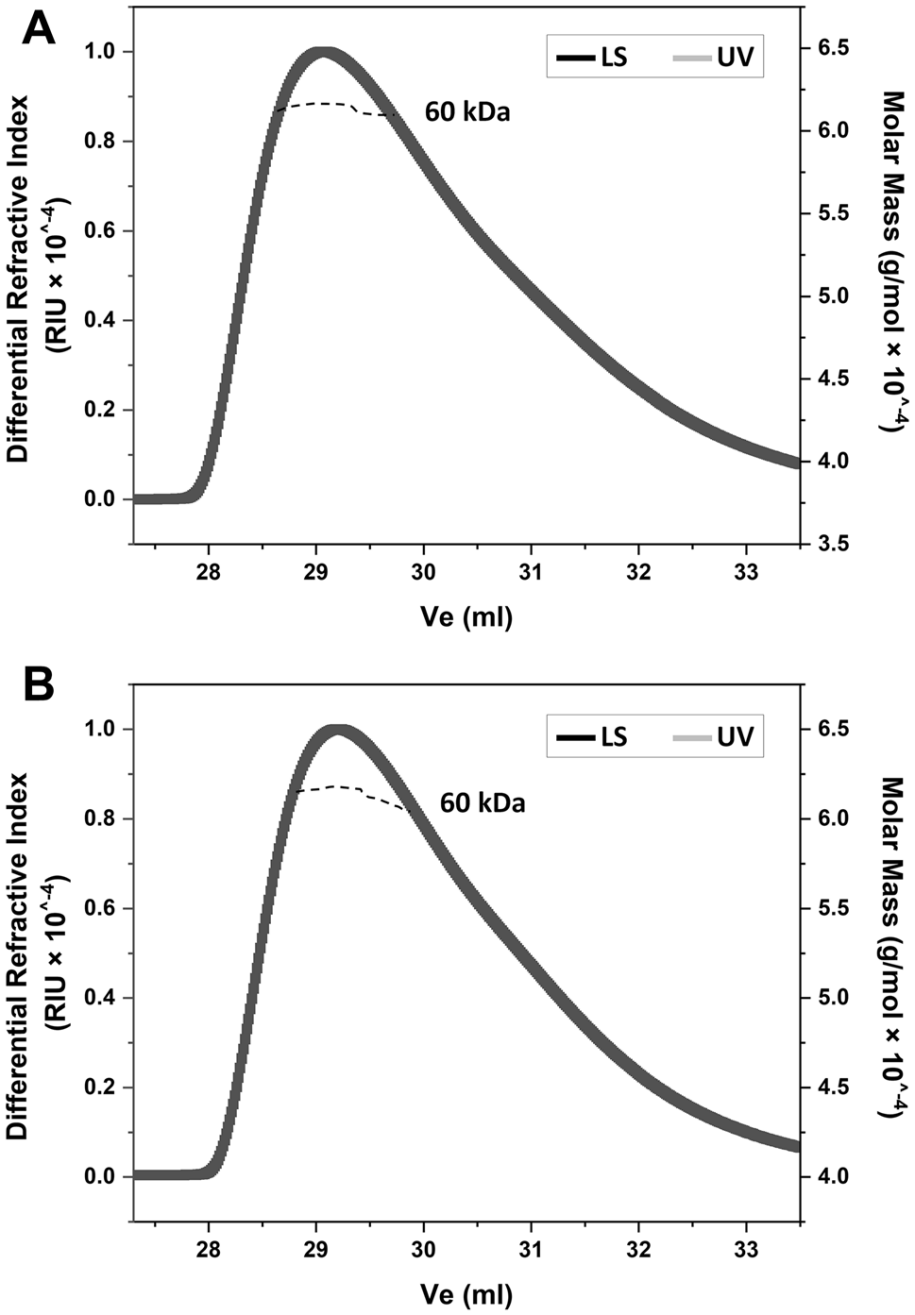
SEC–MALS of BaCcpC. Analysis of the oligomeric state of BaCcpC in the (**A**) presence or (**B**) absence of citrate.

LTTRs are usually functionally active as tetramers and dependent on a coinducer^28^. To verify the oligomeric state of BaCcpC (MW ~ 30 kDa) in solution, we performed size exclusion chromatography coupled to multiangle light scattering (SEC-MALS). BaCcpC-FL formed oligomeric peaks that eluted in the SEC-MALS chromatogram (Fig. 2A). The calculated molecular weights of BaCcpC were ~ 60 kDa (corresponding to 2 monomers). This result indicated that BaCcpC exists as a dimer in the solution. Moreover, in equilibrium buffer with 10 mM citrate, the chromatograms remained dimeric (Fig. 2B). These results indicated that citrate cannot influence the oligomeric state of BaCcpC.

### Overall structure of the IBD of BaCcpC

**Figure 3:**
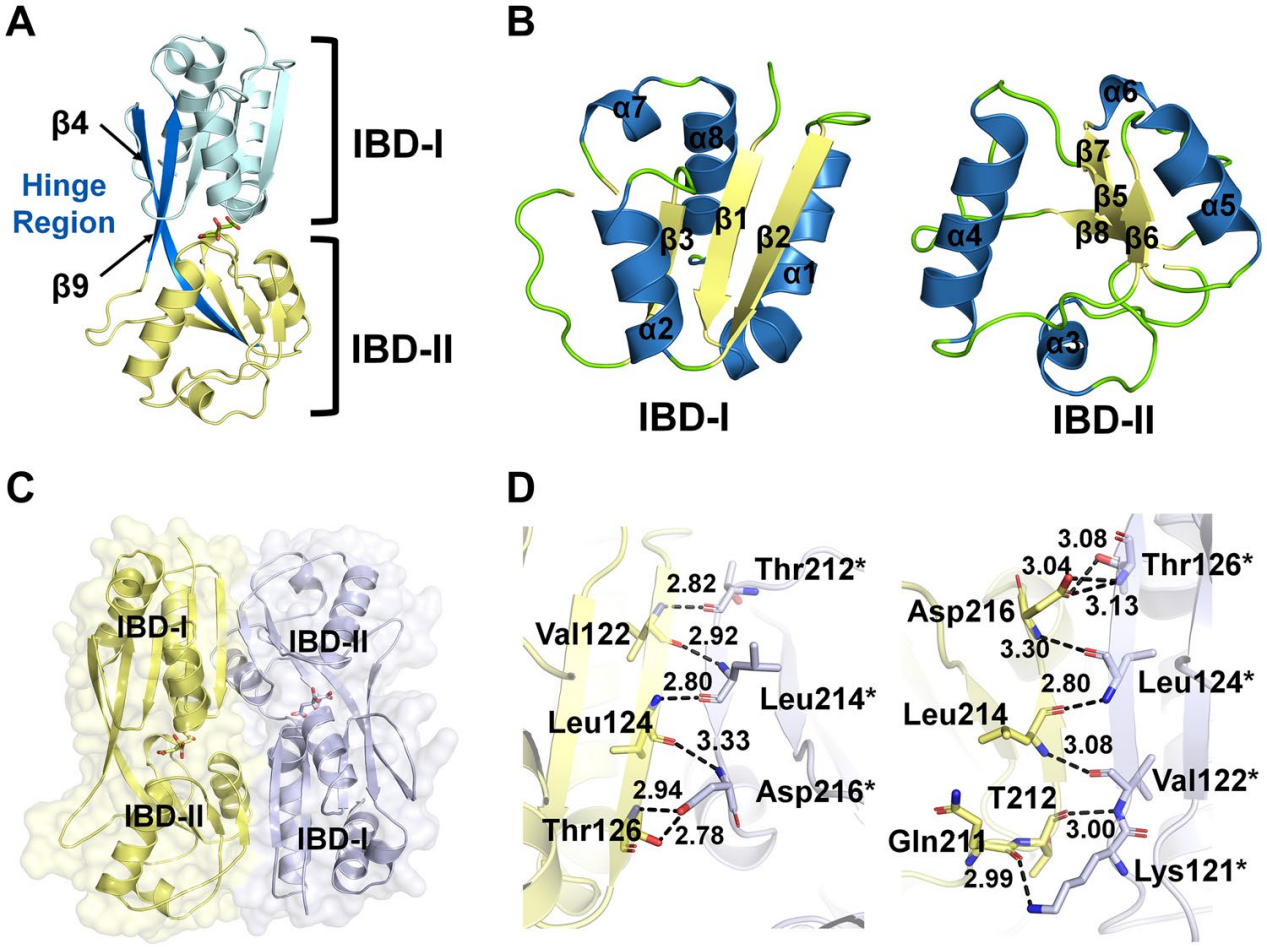
Overall structure of BaCcpC-IBD. (**A**) Monomer structure of BaCcpC-IBD. The hinge regions, consisting of β4- and β9-strands, are indicated in blue. Citrate molecules are located between the IBD-I and IBD-II subdomains. (**B**) Close-up view of the IBD-I and IBD-II subdomains of BaCcpC-IBD. (**C**) Dimer of BaCcpC-IBD. (**D**) Close-up view of the interactions in the dimer interface.

To better understand the molecular function of BaCcpC, we performed a crystallographic study on full-length BaCcpC; however, it was not successful. Furthermore, crystallographic studies for the DBD and IBD of BaCcpC were separately performed. Finally, we obtained crystals for the IBD of BaCcpC and determined the crystal structure of BaCcpC-IBD in complex with citrate at 2.3 Å resolution using single-wavelength anomalous diffraction (SAD) phasing. The BaCcpC-IBD crystal belonged to space group C2 and had unit-cell parameters of *a* = 140.96, *b* = 90.90, *c* = 105.53 Å and β = 106.18°. The *R*_work_ and *R*_free_ of the final model were 20.7% and 26.6%, respectively. The BaCcpC-IBD molecule is composed of two distinct regulatory domains: IBD-I (His90-Arg155 and Gly266-Gln289) and IBD-II (Asp168-Gly259) (Fig. 3A). IBD-I has three β-sheets, which are surrounded by three α-helices and 3_10_ helices (Fig. 3B). IBD-II has four β-sheets, which are surrounded by two α-helices and two 3_10_ helices (Fig. 3B). Both the IBD-I and IBD-II subdomains adopt the typical α/β fold, which is connected by two crossover regions that form a hinge at central regions of two antiparallel β-strands (β4 and β9) (Fig. 3A). Five BaCcpC-IBD molecules are in the asymmetric unit, and each molecule has an RMSD of 0.201–0.275 Å for the 144–180 Cα atoms, which emphasizes the similarity of their conformations. BaCcpC-IBD forms dimers with a head-to-tail arrangement in the asymmetric unit of its crystal structure, and both molecules have essentially the same overall structure (Fig. 3C). Superposition of two dimeric molecules in the asymmetric unit gives an RMSD of 0.327 Å for 321 Cα. The dimeric interface is stabilized by the main chain interactions Val122-Thr212* (2.82 Å, * denoting the partner molecule), Val122-Leu214* (2.92 Å), Leu124-Asp216* (2.80 Å), and Thr126-Asp216* (3.33 Å) between the β2 strand and β6 strand (Fig. 3D).

### The citrate binding site of BaCcpC-IBD

**Figure 4:**
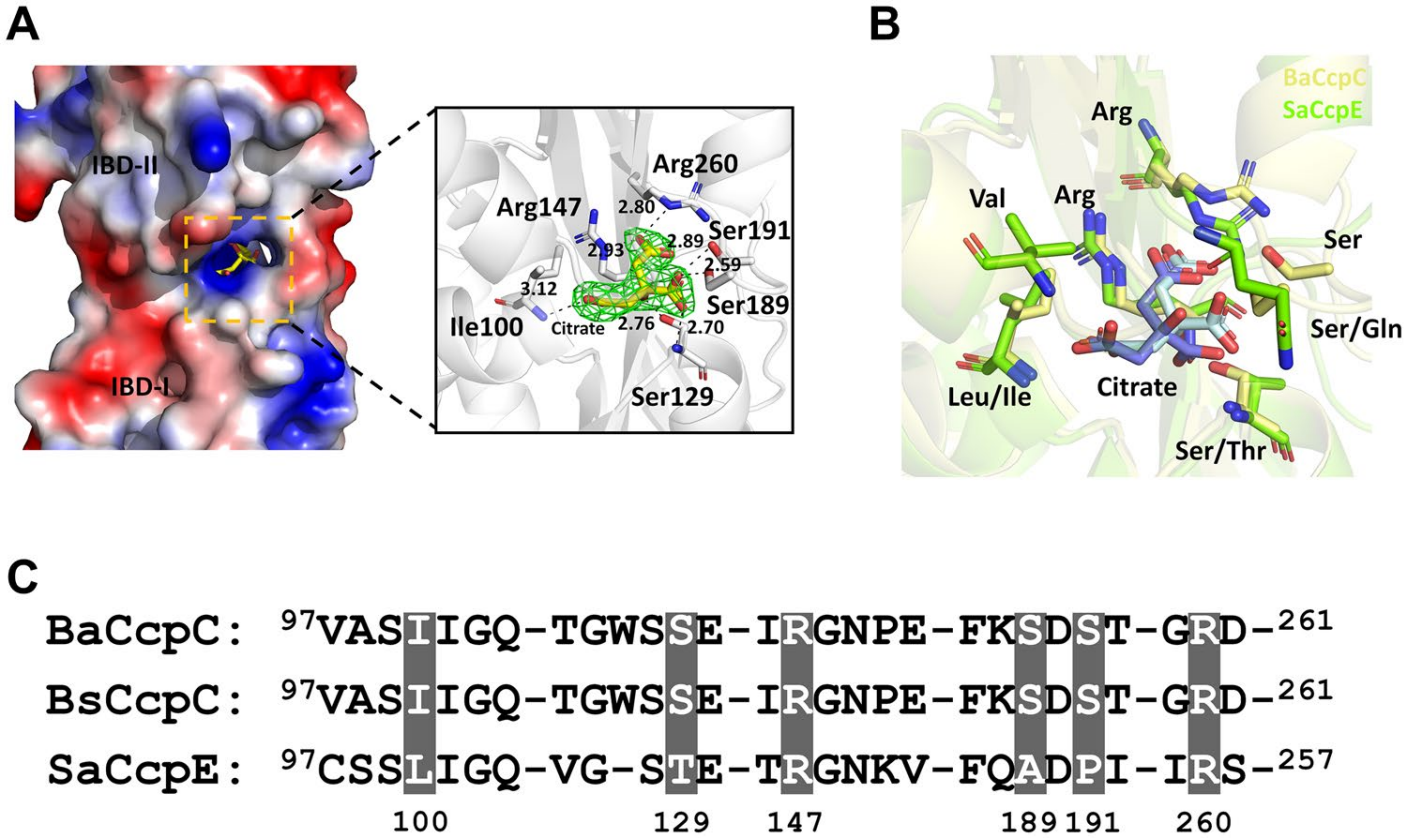
Citrate binding site of BaCcpC-IBD. (**A**) Citrate binding to the interface between IBD-I and II of BaCcpC-IBD. (Insert) Interaction between citrate and BaCcpC-IBD. Simulated annealing 2mFo-DFc omit electron (green mesh, 1σ) density map for citrate molecules. (**B**) Partial sequence alignment of the citrate binding sites of BaCcpC, BsCcpC, and SaCcpE. (**C**) Superimposition of the citrate binding sites of the IBD of BaCcpC (yellow) and SaCcpC (green).

To observe the citrate-bound state of BaCcpC-IBD, we added sodium citrate to the purification buffer during all protein purification steps. The electron density corresponding to citrate molecules is found at the positively charged interface between IBD-I and IBD-II of BaCcpC-IBD (Fig. 4A). In the electron density map, the positions of each carboxyl and hydroxyl group of citrate are clearly distinguished (Fig. 4A). Citrate is a small organic acid that includes three carboxyl groups, one hydroxyl group, and one prochiral centre. To distinguish between the terminal carboxyl groups, these were named *pro-R* and *pro-S*. The *pro-R* carboxyl group accepts a strong hydrogen bond from the backbone nitrogen atom of Ile100 (average distance for five molecules in the asymmetric unit: 3.12 Å). In addition, the pro-S carboxyl group also has hydrogen bonds from the side-chain NE atoms of Arg147 (2.93 Å) and Arg260 (2.80 Å). The central carboxyl group of citrate accepts hydrogen bonds from the backbone nitrogen atom of Ser129 (2.70 Å), the side-chain hydroxyl group, and Ser189 (2.59 Å) and Ser191 (2.89 Å). The hydroxyl group of citrate interacts with the side-chain hydroxyl group of Ser129 (2.76 Å). The atoms of these residues that contact the citrate molecule are ~ 3.0 Å away from the latter’s oxygen atoms, demonstrating that citrate was coordinated by extensive strong hydrogen-bonding interactions (Fig. 4A).

Inducers are important for the function of LTTRs and often participate in the feedback loop of a specific metabolic/synthesis pathway^29^. However, citrate molecules are inducers of BaCcpC, BsCcpC, and SaCcpE^8,9^. Sequence alignment showed that the Arg147 and Arg260 residues of BaCcpC are highly conserved in both BsCcpC and SaCcpE, whereas Ile100, Ser129, Ser189, and Ser191 of BaCcpC are not conserved in SaCcpE and are conserved in only BsCcpC (Fig. 4B). Moreover, the citrate-binding Arg147 and Arg260 residues of BaCcpC-IBD are analogous to the citrate-binding Arg145 and Arg256 residues of CcpE, which are required for CcpE to evoke an appropriate response in the presence of citrate^20,30^ (Fig. 4C). Therefore, we consider these two arginine residues to also play important roles in the citrate binding and functional assembly of BaCcpC.

### Comparison of BaCcpC-IBD with other IBDs from the LTTR family

**Figure 5:**
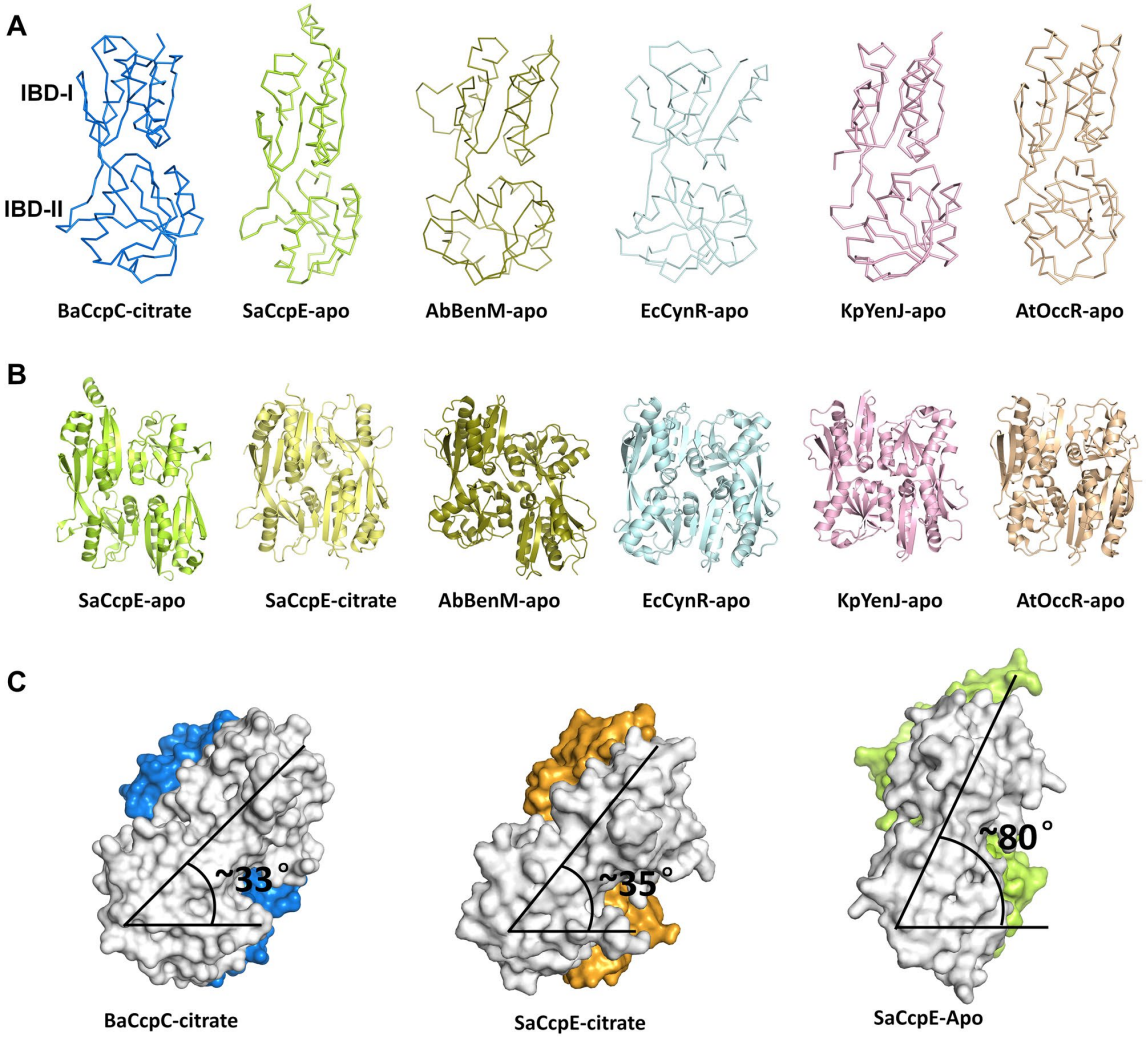
Structural comparison of BaCcpC-IBD with the LTTR family. (**A**) Ribbon representations of the IBDs (or CBDs) of BaCcpC, SaCcpE, AbBenM, EcCynR, KpYneJ and AtOccR, which consist of two subdomains. (**B**) The tail-to-tail dimers arrangement of BaCcpC, SaCcpE, AbBenM, EcCynR, KpYneJ, and AtOccR. (**C**) Comparison of rotation in BaCcpC-IBD-citrate, SaCcpE-IBD-citrate, and SaCcpE-IBD-apo dimers.

To better understand the structural properties of BaCcpC-IBD, its homologues were sought using the Dali server. The IBD of CcpE from *S. aureus* (named SaCcpE-IBD, Z-score: 22.0, sequence identity: 28%, Protein Data Bank (PDB) code: 4QBA), C-terminal domain of a putative transcriptional regulator from *Klebsiella pneumoniae* (KpYneJ-CTD, 20.6, 19%, 5TPI) effector binding domain of BenM from *Acinetobacter baylyi* (AbBenM-EBD, 18.3, 15%, 2F6G), ligand-binding domain of CynR from *Escherichia coli* (EcCynR-LBD, 18.8, 16%, 3HFU) and ligand-binding domain of OccR from *Agrobacterium tumefaciens* (AtOccR-LBD, 19.3, 17%, 5VVH) showed structural similarity to BaCcpC. These proteins belong to the LTTR family. BaCcpC is involved in citrate metabolism, similar to SaCcpE, while AbBenM, EcCynR, AtOccR, and KpYneJ are involved in aromatic compound catabolism, cyanate detoxification, octopine catabolism, and biosynthesis of cysteine, respectively^8,17,28,31,32^. Although BaCcpC-IBD shared low amino acid sequence identities (less than 30%) with SaCcpE-IBD, KpYneJ-CTD, and AtOccR-LBD, it was commonly composed of two subdomains, similar to IBD-I and IBD-II of BaCcpC-IBD (Fig. 5A). The superposition of BaCcpC-IBD with SaCcpE-IBD-apo, SaCcpE-IBD-citrate, KpYneJ-CTD, AbBenM-EBD, EcCynR-LBD, and AtOccR-LBD showed structural similarity with RMSDs of 1.818 Å, 1.181 Å, 2.240 Å, 2.594 Å, 2.315 Å, and 1.941 Å, respectively.

SaCcpE-IBD, AbBenM-EBD, EcCynR-LBD, KpYneJ-CTD, and AtOccR-LBD also show dimers with the same tail-to-tail alignments as BaCcpC-IBD, but the dimeric interfaces and the rotations between these molecules exhibit significant differences. The dimeric interface of SaCcpE-IBD is formed by hydrogen bonds as well as some salt bridges between α1 and α5*, α1 and loop*, and loop and loop* (an asterisk indicates the partner molecule) (Fig. 5B). The dimer interface of AbBenM-EBD consists of the same interaction in α1 and α6* β2-loop*, however, EcCynR-LBD only consists of hydrogen bonds in α1-α5*, β2-β6* β2-loop*. The dimer interface of KpYneJ-CTD consists of two α-helices that interact with one β-sheet, namely, α1-α5*, β2-α5*, α5-β2* and α5-α1*, which differ from the dimer interface of AtOccR-LBD in α1-α5*, loop-loop* and β2-β7* (Fig. 5B and Supplementary Fig. 1). These findings indicated that IBD dimers are relatively stable, even after poor conservation in IBDs. Meanwhile, the rotation angles of the dimers are distinct. In addition, CcpC-IBD is functionally distinct from CcpE-IBD but also recognizes citrate molecules. The monomers of citrate-bound BaCcpC-IBD and SaCcpE-IBD dimers are rotated at angles of approximately 33° and 35°, respectively (Fig. 5C). As a result, the rotation angle of the dimer interface of citrate-bound BaCcpC-IBD is very similar to that of citrate-bound SaCcpE-IBD. On the other hand, in the citrate-free state of SaCcpE-IBD, the angle of the dimer interface is approximately 80°, indicating that there is a change in dimer formation depending on citrate binding. The conformational change between the BaCcpC-IBD domains by the citrate molecule is important for explaining the functional mechanism. To better understand the citrate-induced structural change of BaCcpC, we performed molecular dynamics (MD) simulations. However, there was no significant structural change in BaCcpC with or without citrate molecules (Supplementary Fig. 2). To confirm whether the BaCcpC-IBD domain undergoes a structural change according to the citrate molecule like the CcpE-IBD domain, the apo-state BaCcpC-IBD structure needs to be determined in further studies.

## Conclusion

BaCcpC needs both dyad symmetry regions I and II to recognize the *citB* promoter and the presence of citrate-reduced *citB* binding. Citrate binds the interface between IBD-I and IBD-II of the IBD of BaCcpC. The IBD of BaCcpC shares low sequence similarity with other IBDs of the LTTR family but exhibits similarity in terms of the overall structure and dimer formation. Our results provide the framework for functional analysis of CcpC as well as the diversity and similarity of IBDs of the LTTR family.

## Methods

### Construction, expression, and purification

The full-length (residues 1–293; named BaCcpC-FL) and C-terminal region of the IBD (residues 88–293; named BaCcpC-IBD) of CcpC were obtained from genomic DNA of *B. amyloliquefaciens* by PCR. The gene was cloned into the *Nco*I and *Xho*I sites of the pPROEX-HTA vector (Invitrogen, USA), which contains a hexahistidine tag (MSYYHHHHHH), a spacer region (DYDIPTT) and a tobacco etch virus (TEV) protease cleavage site (ENLYFQ) at the N-terminus. The construct was transformed into *E. coli* BL21 (DE3) competent cells to obtain the target proteins. Protein expression and purification procedures were the same for BaCcpC-FL and BaCcpC-IBD. Cells were grown in 2 L of Luria–Bertani (LB) medium containing 0.5 μg ml^−1^ ampicillin at 310 K. When the OD_600_ of the culture reached 0.8, 0.5 mM isopropyl-β-d-thiogalactoside (IPTG) was added, and the culture was incubated at 303 K for 8 h. The bacterial cells were centrifuged for harvesting and resuspended in lysis buffer containing 20 mM Tris (pH 8.0), 10 mM sodium citrate, 150 mM NaCl, and 2 mM β-mercaptoethanol. Then, the cells were disrupted by sonication, and the lysate was centrifuged at 13,000 rpm for 30 min at 277 K. The lipid fractions were mixed with a nickel-nitrilotriacetic acid (Ni–NTA) affinity resin (GE Healthcare) that had been preincubated with lysis buffer and stirred for 30 min at 277 K. The resin was washed and eluted with lysis buffer containing 20 mM imidazole and 300 mM imidazole. The fractions containing BaCcpC were pooled, and β-mercaptoethanol was added to 10 mM (final concentration). To remove the hexahistidine tag, the mixture was incubated with a recombinant TEV protease at 298 K overnight. For further purification, the mixture was diluted fourfold using 20 mM Tris (pH 8.0) buffer and loaded onto a Q anion-exchange column (HiTrap-Q; GE Healthcare, USA). The fractions containing BaCcpC were purified using a HiLoad Superdex 200 gel filtration column (GE Healthcare, USA) pre-equilibrated with buffer containing 20 mM Tris (pH 8.0), 10 mM sodium citrate, 150 mM NaCl and 2 mM β-mercaptoethanol. To express selenomethionine (Se-Met)-substituted CcpC-IBD protein, the bacterial cells were cultured in 1 L of M9 medium supplemented with an amino acid mixture containing L-( +)-Se-Met at 310 K. When the OD_600_ was between 0.6 and 0.8, the cells were induced with 0.5 mM IPTG for 8 h. The Se-Met-substituted protein was purified under the same conditions as the native protein. To obtain crystal structures of BaCcpC-IBD bound to citrate, we added 10 mM sodium citrate throughout the whole purification process. During purification, the presence of the proteins was detected by sodium dodecyl sulphate–polyacrylamide gel electrophoresis (SDS-PAGE) in a 15% gel with Coomassie blue R-250 for staining.

### Electrophoretic mobility shift assay (EMSA) experiments

A chemiluminescent EMSA kit was purchased from Beyotime Biotechnology (Nanjing, China), and a biotin-labelled *B. amyloliquefaciens citB* promoter was synthesized by Generation (Wuhan, China). Supplementary Table S1 provides the list of oligonucleotide sequences used for EMSA analysis. EMSA experiments between BaCcpC and *citB* promoter DNA were performed at room temperature. For EMSA between BaCcpC and the *citB* protomer, various concentrations of purified BaCcpC (2.5–40 μM) protein were incubated with the the *citB* promoter (800 nM) for 30 min. For EMSA of BaCcpC with regions I and II of the *citB* promoter, purified BaCcpC (40 μM) protein was incubated with each *citB* promoter (800 nM). To determine the effect of citrate on binding between BaCcpC and the *citB* promoter, BaCcpC (40 μM) protein was incubated with *citB* promoter (800 nM) at various citrate concentrations (0–70 μM) for 30 min. After incubation, the reaction mixture was placed in a 6% acrylamide gel on ice using 0.5 × Tris/Borate/EDTA (TBE) buffer. The product was analysed by chemiluminescence detection with the Tanon 4600 Chemiluminescent Imaging system (Tanon, China).

### Size-exclusion chromatography coupled to multiangle light

The oligomer states of BaCcpC-FL were analysed by size-exclusion chromatography coupled to multiangle light scattering. One hundred microlitres of the BaCcpC-FL protein that had been incubated or not incubated with 10 mM sodium citrate was loaded into a Superdex 200 Increase 10/300 GL column (GE Healthcare) at 295 K with a flow rate of 0.5 ml min^−1^ The aqueous mobile phase consisted of 20 mM Tris buffer (pH 8.0) containing 150 mM NaCl and 2 mM 2-mercaptoethanol. Data were collected and processed using ASTRA 6 (Wyatt Technology, USA).

### Crystallization, data collection, structure determination, and refinement

BaCcpC-IBD was concentrated to 20 mg ml^−1^ using a Vivaspin centrifugal concentrator (Cut-off: 10 kDa, Millipore, USA). The initial crystallization was performed using the sitting-drop vapour-diffusion method at 295 K using a Crystal Screen HT high-throughput reagent kit (Hampton Research, USA). Crystals of BaCcpC-IBD were grown in 10% polyethylene glycol 6000, 5% 2 methyl-2,4-pentanediol (MPD), and 0.1 M HEPES (pH 7.5) using a 1:1 ratio of protein to mother liquor at 287 K. Finally, crystals of BaCcpC-IBD were obtained in sitting drops over 8% (w/v) polyethylene glycol 6000, 6% MPD, and 0.1 M HEPES (pH 7.5) using a 1:1 ratio of protein to mother liquor at 287 K. Immediately after the single crystals were taken from their drop, they were soaked for 5 s in cryoprotectant solution consisting of the mother liquor solution containing 25% (v/v) glycerol and subsequently flash-cooled in liquid nitrogen.

The dataset was collected at 100 K using an ADSC Q310 CCD detector at Beamline 7A, Pohang Accelerator Laboratory (Pohang, Republic of Korea). The peak wavelength of Se-Met in BaCcpC was determined to be 0.9826 Å by a fluorescence scan. Data were collected using the inverse beam method with an oscillation range of 1° per frame over a 360° rotation, and the exposure time was 5 s per frame. The crystal of the BaCcpC-IBD protein was diffracted to 2.3 Å resolution. The diffraction data were processed, merged, and scaled using the *HKL*-2000 program^33^. Initial phases were obtained using AUTOSOL in the software package PHENIX 1.15.2_3472 (phenix-online.org)^34^. Refinement was performed using the Crystallographic Object-Oriented Toolkit (COOT) and phenix.refine^35,36^. The data collection and refinement statistics are given in Table 1. Structural images were generated by PyMOL^37^.

**Table 1:** Crystallography data and refinement statistics.

**Data sets**	BaCcpC-IBD
Beamline	Beamline 7A at PLS
Resolution range (Å)	29.71–2.30
Space group	C2
Total /unique reflections	55,744/4795
*a, b, c* (Å)	140.96, 105.53, 106.19
R_sym_ (%)	8.0 (3.1)
Completeness (%)	96.46 (83.04)
Multiplicity	5.3 (2.6)
*I/σ(I)*	36.2 (2.64)
**Model refinement**	
^b^R_factor_ /R_free_ (%)	20.75 (34.77)/26.70 (41.22)
No. of protein atoms	7976
No. of water molecule	153
Average B factor (Å^2^)	62.00
R.m.s.d (Bond)	0.008
R.m.s.d (Angles)	0.94
**Ramachandran plot (%)**	
Favored regions	95.53
Allowed regions	4.16
Disalowed regions	0.31
PDB code	7DMW

^a^Values in the parentheses refers to the highest resolution shell.

^b^∑hkl│Fo-Fc│/∑hkl│Fo│ for all data with Fo > 2σ(Fo), excluding data used to calculate Rfree. §Rfree = ∑hkl│Fo-Fc│/∑hkl│Fo│ for all data with Fo > 2σ (Fo) that were excluded from refinement.

### Molecular dynamics

Simulations of the CcpC-IBD domain (apo state of monomer and apo state of dimer) were performed using the GROMACS GROMACS 2020.3 manual (www.gromacs.org)^38^. For the simulation, the Amber99sb-ildn force field and explicit solvent based on the TIP3P model were employed^39,40^. The structures were solvated with explicit water in periodic rectangular boxes under normal (150 mM) saline conditions. The LINCS algorithm was used to constrain all bond lengths^41^. Long-range electrostatic interactions were treated with the particle-mesh Ewald method^40^. For the MD calculations, the nonbonded (electrostatic and VDW) cut-off range was 10 Å and the time step was 2 fs.

Before each MD simulation, the entire system was first minimized by a 1000-step steepest descent calculation followed by a 50,000-step conjugate gradient optimization. The total computer simulation time for BaCcpC-IBD was 1 ns.

## Supplementary information

Supplementary Information.

## Data Availability

The atomic coordinates and structure factors for BaCcpC-IBD (PDB ID 7DMW) have been deposited in the RCSB Protein Data Bank (www.rcsb.org).
