# Supplementary material for: Functional and structural analysis of catabolite control protein C that responds to citrate
Source: Sci Rep. 2021 Oct 13;11:20285. doi: 10.1038/s41598-021-99552-x (PMC8514465; doi:10.1038/s41598-021-99552-x)
Supplement: Supplementary file 1 — Supplementary Information. [file 41598_2021_99552_MOESM1_ESM.docx]

**Supplemental Information**

**Functional and structural analysis of catabolite control protein C that responds to citrate**

Wei Liu ^1,2†^, Jinli Chen ^3†^, Liming Jin^1,2^, Zi-Yong Liu ^4^, Ming Lu ^4^, Ge Jiang ^5^, Qing Yang ^3^, ChunshanQuan^1,2*^, Ki Hyun Nam ^6*^, and Yongbin Xu ^1,2*^

^1^ Department of Bioengineering, College of Life Science, Dalian Minzu University, Dalian 116600, Liaoning, China

^2^ Key Laboratory of Biotechnology and Bioresources Utilization of Ministry of Education, College of Life Science, Dalian Minzu University, China
^3^ School of Life Science and Biotechnology, Dalian University of Technology, No. 2 Linggong Road, Dalian 116024, Liaoning, China

^4^ Shandong Provincial Key Laboratory of Energy Genetics, key Laboratory of Biofuel, Qingdao Institute of Bioenergy and Bioprocess Technology, Chinese Academy of Sciences, Qingdao 266101, Shandong, China

^5^ School of Life Science and Biotechnology, Dalian University, Dalian 116622, Liaoning, China

^6^ Department of Life Science, Pohang University of Science and Technology, 35398 Pohang, Republic of Korea

*Corresponding author: Tel. (+86) 411 8765 6045; Fax. (+86) 411 8765 6219; E-mail: yongbinxu@dlnu.edu.cn (Y. Xu). Tel. (+82) 10 5208 5730; E-mail: structures@postech.ac.kr (K. H. Nam). Tel. (+86) 411 8765 6219; E-mail: mikyeken@dlnu.edu.cn (C. Quan).

^†^ Both authors contributed equally to this work.

**
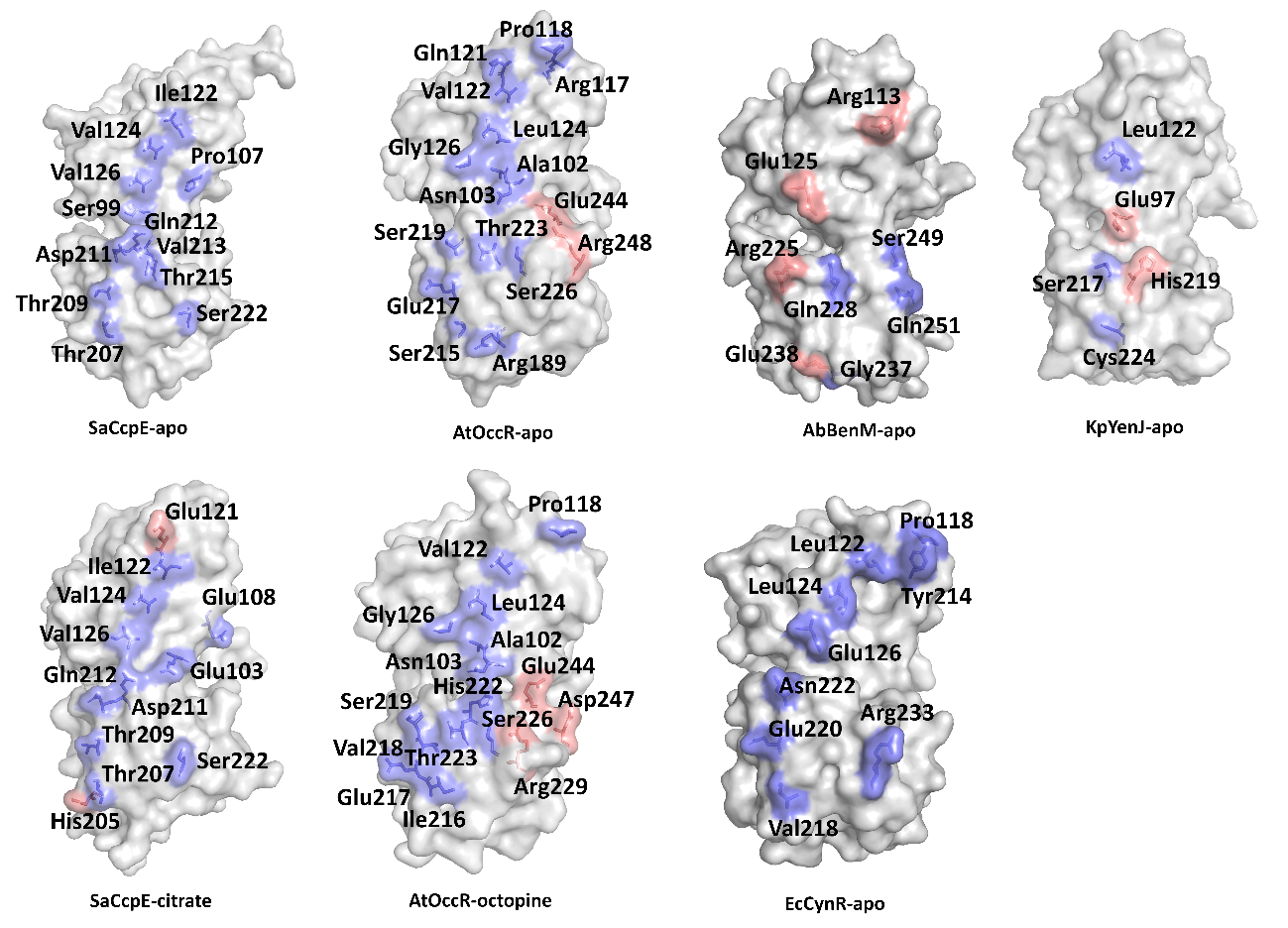
.**

**Supplementary Figure 1.** Dimer interface of IBD domain of SaCcpE (PDB code: 4QBA), AtOccR (5VVH), AbBenM (2F6G), KpYenJ (5TPI), SaCcpE-citrate (5ZZO), AtOccR-octopine (5VVI) and EcCynR (3HFU). Hydrogen-bond and salt bridge interaction between dimer interface are indicated by blue and red surfaces, respectively.


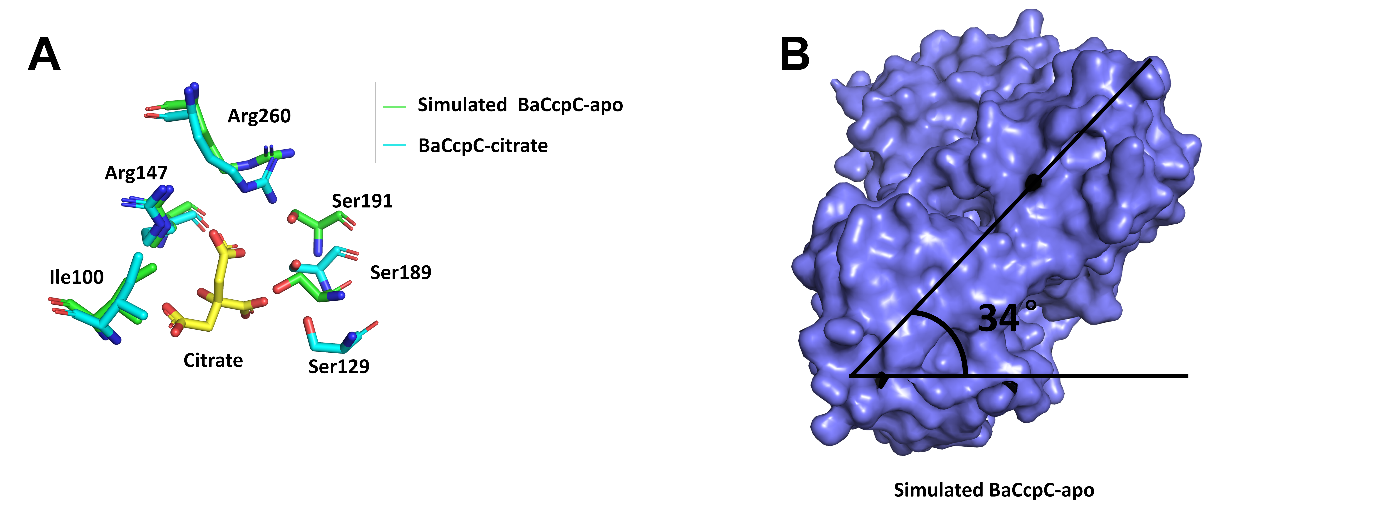


**Supplementary Figure 2.** MD simulation of BaCcpC-apo. (A) Structural comparison of MD simulated-BaCcpC-apo with citrate-bound BaCcpC. (B) Rotation in MD simulated BaCcpC-apo dimer.

**Table S1. Oligonucleotide sequences used for EMSA analysis.**

| *citB*-P | TGATAAGACGATCTTATTGTATTTAATAAAAACATTGATATTTACTTATGTATGAT |
| --- | --- |
| *citB*-PI | TGATAAGACGATCTTATTGT |
| *citB*-PII | TGATATTTACTTATGTATGAT |

**Table S2. Crystallography data and refinement statistics**

| **Data sets**BaCcpC-IBD | |
| --- | --- |
| **Beamline** | Beamline 7A at PLS |
| **Resolution range (Å)** | 29.71-2.30(2.37-2.30) |
| **Space group** | C2 |
| **Total /unique reflections** | 55744/4795 |
| ***a, b, c* (****Å)** | 140.96, 105.53, 106.19 |
| **R_sym_(%)** | 8.0 (3.1) |
| **Completeness (%)** | 96.46 (83.04) |
| **Multiplicity** | 5.3 (2.6) |
| **Average *I/σ(I)*** | 36.2 (2.6) |
| **Model refinement** | |
| **R_factor_/ R_free_(%)** | 20.75 (34.77)/26.70(41.22) 16.47(18.81)/22.53(26.03) |
| **No. of protein atoms** | 7976 |
| **No. of water molecule** | 153 |
| **Average B factor (Å^2^)** | 62.00 |
| **R.m.s.d (Bond)** | 0.008 |
| **R.m.s.d (Angles)** | 0.94 |
| **Ramachandran plot(%)**  **plot(%**  **)**  **PLOTPLOTPreferred (%)** |  |
| **Favored regions** | 95.53 |
| **Allowed regions** | 4.16 |
| **Disalowed regions** | 0.31 |
| **PDB code** | 7DMW |

Values in parentheses are for the outermost shell.
